# Composition and Functional Characterization of Microbiome Associated with Mucus of the Coral *Fungia echinata* Collected from Andaman Sea

**DOI:** 10.3389/fmicb.2016.00936

**Published:** 2016-06-16

**Authors:** Jhasketan Badhai, Tarini S. Ghosh, Subrata K. Das

**Affiliations:** ^1^Department of Biotechnology, Institute of Life SciencesBhubaneswar, India; ^2^Computational and Systems Biology Group, Genome Institute of SingaporeSingapore, Singapore

**Keywords:** coral, Andaman Sea, metagenome, microbial composition, functional analysis

## Abstract

This study describes the community composition and functions of the microbiome associated with the mucus of the coral *Fungia echinata* based on metagenomic approach. Metagenome sequence data showed a dominance of the class *Gammaproteobacteria* followed by *Alphaproteobacteria, Betaproteobacteria, Deltaproteobacteria, Flavobacteriia, Bacilli*, and *Clostridia.* At the order level, the most abundant groups were *Pseudomonadales*, *Oceanospirillales, Alteromonadales, and Rhodobacterales*. The genus *Psychrobacter* was the most predominant followed by *Thalassolituus* and *Cobetia*, although other genera were also present, such as *Sulfitobacter, Pseudoalteromonas*, *Oleispira*, *Halomonas*, *Oceanobacter*, *Acinetobacter*, *Pseudomonas*, *Vibrio*, and *Marinobacter*. The metabolic profile of the bacterial community displayed high prevalence of genes associated with core-housekeeping processes, such as carbohydrates, amino acids, proteins, and nucleic acid metabolism. Further, high abundance of genes coding for DNA replication and repair, stress response, and virulence factors in the metagenome suggested acquisition of specific environmental adaptation by the microbiota. Comparative analysis with other coral metagenome exhibits marked differences at the taxonomical and functional level. This study suggests the bacterial community compositions are influenced by the specific coral micro-niche and the oligotrophic marine environment.

## Introduction

India has a vast reserve of coral reef ecosystem in the east coast of Andaman and Nicobar Islands. Microbial communities are vital in the functioning of all ecosystems; however, most microorganisms are uncultivated, and their roles in natural systems are unclear. In this regard, microbial diversity in the Andaman Sea is one of the least studied among marine environments. In the recent past, metagenomic study has been employed in the field of marine microbial ecology to explore the diversity of unknown microbes in different marine habitats. It has been demonstrated by comparative metagenomic study that functional attributes in the microbial communities are evolved by typical environmental adaptation (Tyson et al., 2004; Daniel, 2005; Simon and Daniel, 2011). Bacteria are known to be abundant in seawater around coral zones, in coral skeleton, tissues, and surface mucus layer (Rohwer et al., 2002; Lampert et al., 2006, 2008; Kooperman et al., 2007; Rosenberg et al., 2007; Bayer et al., 2013), and each of these habitat supports different bacterial species (Koren and Rosenberg, 2006; Littman et al., 2009). However, functional role of microbiome associated with coral is poorly understood, although few studies have suggested their involvement in the supply of nutrients (Lesser et al., 2007; Olson et al., 2009) and inhibition of coral diseases by the production of antibiotics (Rohwer et al., 2002; Ritchie, 2006; Krediet et al., 2013). Metagenomic approach gives a description of the taxonomic information (Tyson et al., 2004), the relative abundance of phylotypes and genes (Wegley et al., 2007; Littman et al., 2011), antibiotic resistance reservoir (Sommer et al., 2009), and identification of genes involved in various biosynthetic and metabolic pathways (Schirmer et al., 2005; Tringe et al., 2005; Rodriguez-Brito et al., 2006). This approach enables assessment of taxonomic and functional characteristics of complex microbial communities.

Coral mucus is a rich source of nutrients for microorganisms (Wild et al., 2004; Brown and Bythell, 2005; Tremblay et al., 2011) and as such the mucus layer constitutes an important ecological niche for microbiome (Lampert et al., 2006, 2008; Nithyanand and Pandian, 2009). Following culture-dependent approach, we have identified several new species of bacteria with unique functions from the corals collected from Andaman Sea (Badhai et al., 2013; Kumari et al., 2014; Lepcha et al., 2015). However, taxonomic and functional profiling of the microbiome of the coral *Fungia echinata* from Andaman Sea has not been performed so far. As the microbial diversity and functions are directly linked with local environment, it is justifying examination of new niches for novel genes and species. In this regard, our metagenomic study with the coral mucus of *F. echinata* provides distinct taxonomical and functional attributes that has not been reported earlier in other marine environment.

## Materials and Methods

### Collection of the Metagenome and Sequencing

**FIGURE 1:**
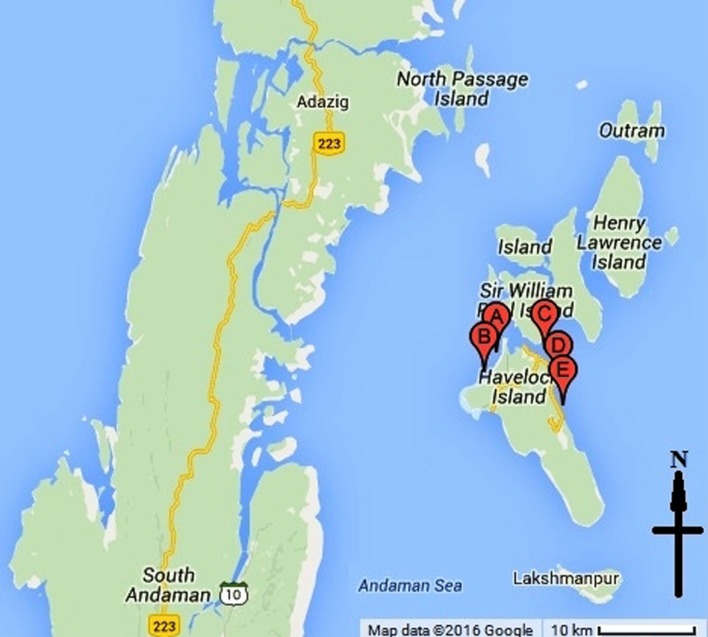
**Location of the sampling sites around the Havelock Island in Andaman Sea.** Points A, B, C, D, and E on the map represent the five sampling sites.

Metagenomic DNA was prepared from the mucus of multiple specimens (five numbers) of coral *F. echinata* collected from five different locations within the reefs around the Havelock Island in Andaman Sea (**Figure 1**). Coral specimens were collected during March, 2012 from a depth of about 3.5–5 m. Metagenomic DNA was prepared from pooled mucus samples following an earlier protocol (Goldenberger et al., 1995) with slight modifications. Typically, the metagenome extraction process starts by filtering samples onto 0.22-μm Millex filters. In this regard, corals were briefly expose to air (Wild et al., 2005), placed in a sterile tray and then repeatedly flushed with sterile marine water using a 50 ml syringe. About 500 ml of the pooled coral mucus suspension in marine water was filtered and the membrane was collected and washed with 10 ml Milli-Q water by vortexing. The suspension was centrifuged at 14,000 rpm for 10 min to collect the cell pellet. Cell pellet was transferred to a Lysing Matrix E tube. Reaming procedures was followed as per manufacturer instruction (FastDNA^®^ SPIN Kit for Soil, MP Biomedicals, CA, USA). The concentration of DNA was approximately 150 ng μl^-1^. Sequencing of the metagenomic DNA samples were done by a commercial source (NxGenBio Life Sciences, New Delhi, India) applying shotgun pyrosequencing approach on a Roche 454 GS-FLX platform (Roche Applied Sciences, Manheim, Germany) according to the manufacturer’s protocol. A total of 40,938 reads representing cumulative 20.61 Mb of sequence data were obtained.

### Metagenomic DNA Sequence Data Generation

Raw sequence reads were processed and calculation of DNA sequence statistics such as length and GC content of the processed reads were carried out using the freely available online WebMGA server^1^ (Wu et al., 2011). The total shotgun metagenomic sequences from each site were preprocessed using the following parameters: (i) quality filtration (at min length = 200 bases and min average quality score = 21) and (ii) CD-HIT-454 clustering at a sequence identity threshold of 0.98 to remove artificial duplicates generated during sequencing (Niu et al., 2010). Subsequently, after the preprocessing approx. 26,534 sequence reads (av. sequence length = 516 bases, av. % GC content = 48.73) were retained for further analysis.

### Taxonomic and Functional Analysis of Metagenome

Taxonomic and functional assignments for the protein-coding sequences in each metagenomic reads dataset were obtained using BLASTX (Altschul et al., 1997; applying an *e*-value cut-off of 1e–10) against NCBI-NR database (local BLAST-2.2.29+ package; BLAST^®^ Help, 2008; Camacho et al., 2009) and the standalone MEtaGenome analyzer software (MEGAN v5.5.3; Huson et al., 2011) according to suggested parameters for the lowest common ancestor (LCA) assignment algorithm (min score: 50.0; max expected: 0.01; top percent: 10.0; and min support percent: 0.01). To perform taxonomic classification, the MEGAN5 program placed the reads/genes onto the NCBI taxonomy tree, whereas for the functional classifications, genes were mapped onto the SEED and KEGG classification using SEED and KEGG identifiers, respectively. Additionally, taxonomic and functional annotation, and comparisons with other coral metagenomes (Dinsdale et al., 2008; Vega Thurber et al., 2009; Littman et al., 2011) were carried out on the MG-RAST v3.6 metagenome analysis server^2^ (Meyer et al., 2008) using the default set parameters (*e*-value cutoff: < 1e–5, min. % identity cutoff: 60%, and min. alignment length cutoff: 15).

### Statistical Analysis of Data

Comparative taxonomic and functional profiling were performed with the reference coral metagenomes available in MG-RAST database using the STAMP v2.0.8 software (Parks and Beiko, 2010; Parks et al., 2014) for statistical analyses. The gene counts were normalized by dividing the number of gene hits to individual taxa/function by total number of gene hits in each metagenome dataset to remove bias due to difference in sequencing efforts. To identify differentially abundant SEED or KEGG functions in the metagenome, statistical tests of the relative gene abundances compared to the other coral metagenomes were carried out by applying two-sided Welch’s exact test with Benjamini–Hochberg False Discovery Rate (FDR) multiple test correction method and a *p*-value < 0.05.

### Sequence Data Availability

Metagenomic sequence dataset obtained from the mucus of coral *F. echinata* is available on the MG-RAST server under accession ID 4653307.3. The other 12 metagenomes used in the comparative analysis can be accessed through the MG-RAST website under the accession IDs: 4440037.3, 4440039.3, 4440041.3, 4440279.3, 4445755.3, 4445756.3, 4440372.3, 4440373.3, 4440378.3, 4440379.3, 4440380.3, and 4440381.3.

## Results and Discussions

### Taxonomic Composition of the Microbial Community

**FIGURE 2:**
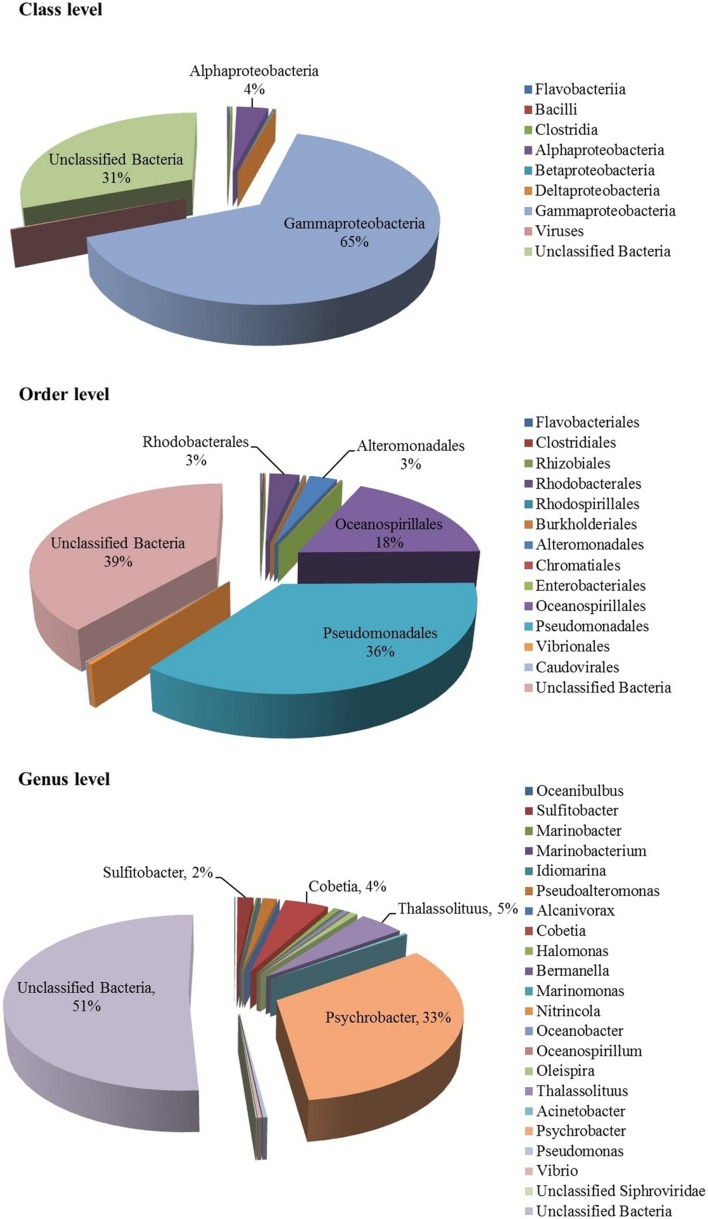
**Taxonomic composition of the mucus-associated microbiome of coral *F*. *echinata* from Andaman Sea based on metagenomic sequencing**.

*Bacteria* constituted the single largest kingdom within the mucus-associated microbial assemblage of the coral *F. echinata*. Approximately 75.96% of the metagenomic sequences were classified as *Bacteria* and only 0.21% as viruses; the remaining 23.83% of the sequences could not be classified due to lack of reference sequences from close taxonomic relatives. The bacterial community in the coral mucus was dominated by sequences affiliated to the class *Gammaproteobacteria* (64.7%), followed by the class *Alphaproteobacteria* (3.5%); the other bacterial sequences (abundances <0.1–0.2%) were affiliated to the *Betaproteobacteria*, *Flavobacteriia*, *Clostridia*, *Deltaproteobacteria*, and *Bacilli* (**Figure 2**). The virus-like sequences were classified as dsDNA viruses of the order *Caudovirales*. *Gammaproteobacteria* was the sole dominant phylum. This phylum displayed a large phylogenetic diversity that might explain the colonization of a large range of ecological environment (Williams et al., 2010). Our analysis of the microbiome of *F. echinata* supported the fact that microbial diversity is directly linked with local environment. Further, this community was different from those reported in earlier studies, which suggested that microbial association with coral species reveal species-specific distribution and geographic variability (Rohwer et al., 2002; Lampert et al., 2006, 2008; Wegley et al., 2007; Littman et al., 2009; Morrow et al., 2012).

At the order level (**Figure 2**), the major bacterial groups identified were *Pseudomonadales* (35.6%), *Oceanospirillales* (17.9%), *Rhodobacterales* (3.0%), and *Alteromonadales* (2.8%), whereas the abundance of taxa (< 1%) were *Vibrionales*, *Clostridales*, *Flavobacteriales*, *Rhodospirillales*, *Burkholderiales*, *Enterobacteriales*, *Chromatiales*, and *Rhizobiales*. Further classification at the genus level suggested that the *Psychrobacter* (32.7%) was the most predominant bacterial group in the metagenome and the other genera that were identified include *Thalassolituus* (4.6%), *Cobetia* (4.5%), *Sulfitobacter* (1.6%), and *Pseudoalteromonas* (1.4%). In addition, less abundant (<1%) genera were also observed (**Figure 2**). The previous study with the metagenome of the coral *Ctenactis echinata* from Red Sea (Roder et al., 2015) demonstrated the predominance of the genus *Endozoicomonas.* In contrast, a significant change was observed in the microbial community of *F. echinata* from Andaman Sea, which become dominated by *Psychrobacter*. As we found that mucus microbiome become more dissimilar from other studies with geographical distance, indicating that different regions harbor distinct communities (Sunagawa et al., 2009; Littman et al., 2010). One possible explanation could be water temperature in the Andaman Sea (25–28°C), leading to the development of *Psychrobacter* community in the mucus layer, which has been frequently identified in the Antarctic deep waters (López-García et al., 2001) and it is more dissimilar with the bacterial communities of fungid coral *C. echinata* in Red Sea (Roder et al., 2015). These differences might be in part due to the physicochemical condition of the Red Sea which has been characterized as an oligotrophic environment with year round UV irradiation and high surface temperature up to 34°C (Ngugi et al., 2012). Further, bacteria particularly *Vibrio* species are ubiquitous have been implicated as the causative agent in some cases of coral and influencing the coral health (Rosenberg et al., 2007; Bourne et al., 2008), but very low abundance of sequences affiliated to the *Vibrio* group probably due to specific micro-niche (i.e., surface mucus layer) or the general structure of the bacteria–coral association. The unclassified group representing 23.83% of the sequences may serve important functional roles in biogeochemical cycle and degradation of xenobiotic compounds and warrant further investigation, especially in highly dynamic ecosystem.

### Functional Analysis of the Metagenome

**FIGURE 3:**
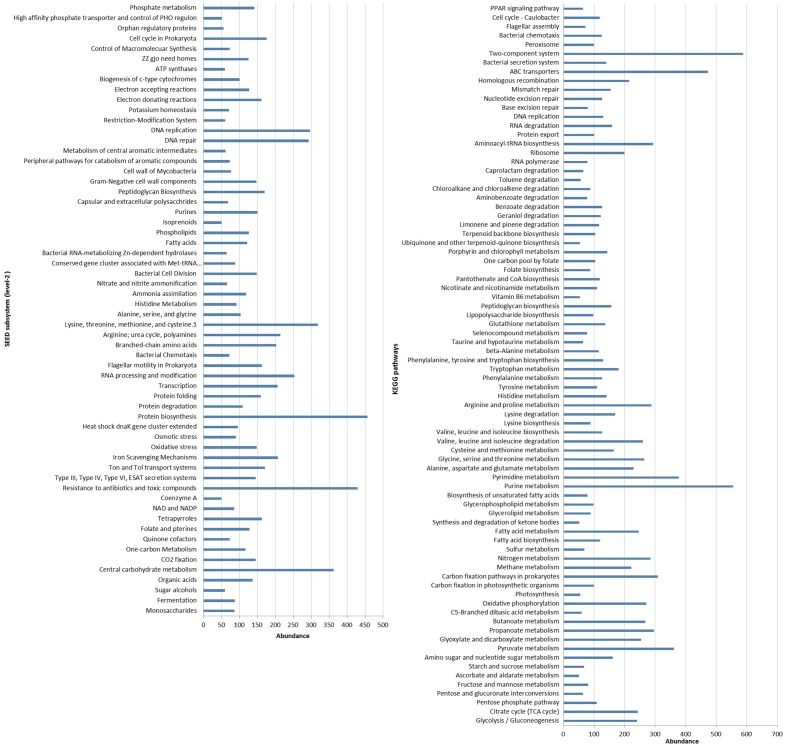
**Distribution of functional genes related to various SEED subsystems (level 2) and KEGG pathways present in the metagenome of the coral *F. echinata*.** Only genes with an abundance of at least 50 are shown.

Functional analysis of the metagenomic showed approximately 60 and 36% of the predicted protein coding sequences or genes were matched to 25 SEED subsystems and 23 KEGG categories, respectively (**Figure 3**). Analysis of metagenome suggested that genes associated with the core-housekeeping functions, such as carbohydrates, amino acids, proteins, nucleotides, cofactors, and vitamin metabolism were the most abundant metabolic categories (**Supplementary Figure S1**). Our result corroborated the findings on coral-associated microbiomes in tropical environments (Wegley et al., 2007; Tout et al., 2014). In addition, genes affiliated to virulence, stress response, signal transduction, membrane transport, and DNA replication and repair functions were also highly abundant in our dataset (**Supplementary Figure S1**).

The predicted genes encoding virulence functions (SEED level 2), such as iron-scavenging mechanisms, Ton and Tol transport systems, and protein secretion systems, were highly abundant (**Supplementary Figure S2**). As iron is an important micronutrient and poorly available in the seawater (Wells et al., 1994), the metabolic ability to take up and store iron is advantageous and these functional subsystems may have a modulatory role in the structuring of the coral-associated bacterial community. Further, genes encoding resistance to antibiotics and toxic compounds along with the oxidative stress, and DNA replication and repair functions were also highly represented (**Figure 3**, **Supplementary Figure S2**) and predicted that these coral-associated bacteria are more suited to deal with intense selection pressure and environmental stressors, such as heavy metal ions, reactive oxygen, and ultraviolet radiations, etc. The abundance of genes associated with the metabolic functions such as bacterial chemotaxis, two-component systems and ABC transporters (**Figure 3**) predicted higher level of cellular interactions and metabolic exchanges between the bacterial community and coral host (Tout et al., 2014; Ainsworth et al., 2015).

**FIGURE 4:**
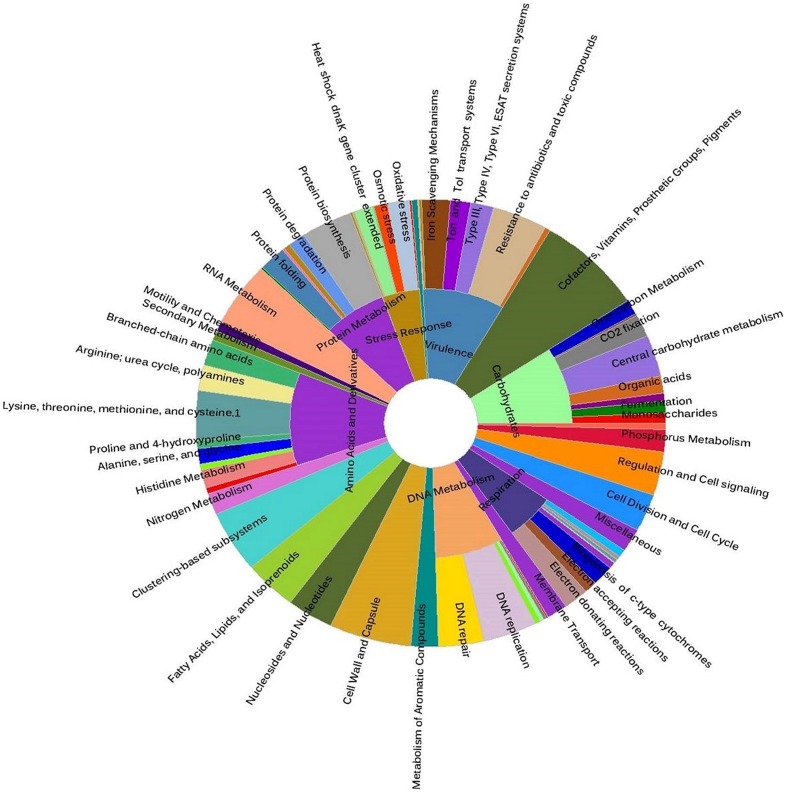
**Metabolic profile of the mucus-associated bacterial genus *Psychrobacter*, based on SEED subsystem classification of the metagenomic sequences obtained in this study**.

Earlier, McKew et al. (2012) reported the abundance of *Psychrobacter* in the mucus of *Acropora* spp. and *Porites* spp., but they have not analyzed the functions of these bacteria in the coral holobiont. In this regard, our analysis of genes affiliated to the genus *Psychrobacter* identified their functional role in the carbon and nitrogen metabolism within the coral niche. Accordingly, the abundance of genes related to carbohydrates, lipids and amino acids metabolisms (**Figure 4**) indicated the high metabolic potential of these bacteria to efficiently utilize the complex organic compounds in the coral mucus. Further, presence of the *qseC* gene predicted these bacteria respond to quorum sensing and form biofilm-like structure to successfully colonize the coral surface (Yang et al., 2014). In addition, the presence of genes corresponding to the two-component systems for sensing phosphate limitation (*phoR*, *phoB*, and *phoD*), stress (*rstA* and *rstB*), copper tolerance (*cusS* and *cusR*), nitrate respiration (*narX* and *narL*), and low nitrogen availability (*glnL* and *glnG*) revealed that these bacteria are better adapted to the continuous chemical fluctuations in the seawater surrounding the coral niche. Of the other metabolic categories that increased significantly within the coral metagenome, it is notable that genes associated with ABC transporters for ions of sulfate (*cysPUWA*), phosphate (*pstS*, *pstC*, and *pstB*), phosphonate (*phnD*), zinc (*znuABC*), nickel (*oppABCDF*), and iron (*afuABC* and *fhuBCD*). Furthermore, genes homologous to virulence, stress response, and DNA replication and repair functions were examined (**Figure 4**). Taken together, it appears that microorganisms associated with the mucus of the coral *F. echinata* have mechanisms to deal with biotic or abiotic stressors within the coral niche and help in their colonization.

### Comparative Analysis of Metagenomic Sequences with MG-RAST DataBase

The taxonomic and functional profiles of mucus-associated microbiome of *F. echinata* was compared with the microbial communities associated with (i) colonies of coral *Acropora millepora* from the Great Barrier Reef (Littman et al., 2011), (ii) coral *Porites compressa* from Hawaii (Vega Thurber et al., 2009), and (iii) four coral atolls (Kingman, Kiritimati, Palmyra, and Tabuaeran) in the Northern Line Islands, central Pacific (Dinsdale et al., 2008).

**FIGURE 5:**
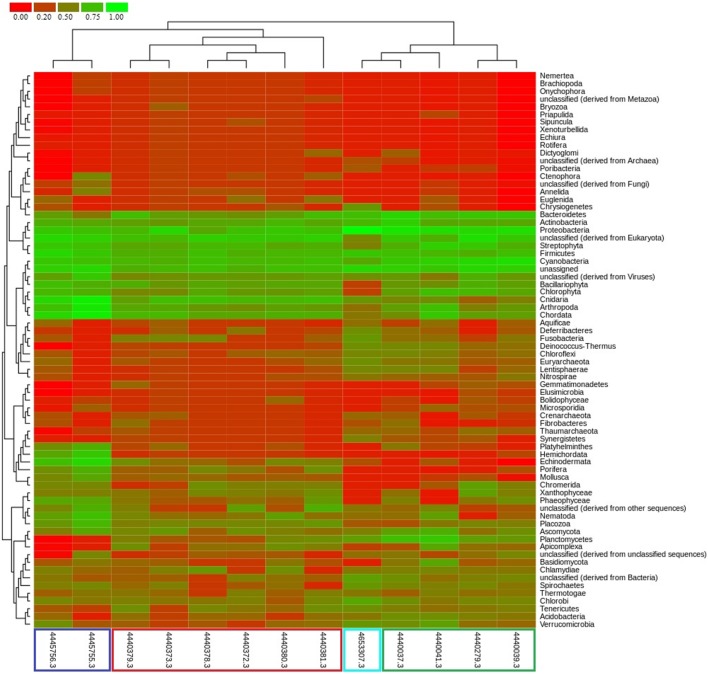
**Comparative taxonomic profile of metagenomes generated using MG-RAST based on best hits to M5NR database.** Metagenome data source: this study (cyan color), Dinsdale et al., 2008 (green color), Vega Thurber et al., 2009 (red color), and Littman et al., 2011 (blue color).

The taxonomic profile of microbial community associated with the mucus of *F. echinata* showed an exclusive dominance of *Bacteria*, belonging to the phylum *Proteobacteria* (**Figure 5**), whereas the functional profile showed higher abundance of genes involved in core-housekeeping functions, as well as genes encoding specialized and ecologically important metabolic functions. These genes are related to the metabolisms of glutathione, biotin, riboflavin, butanoate, tyrosine, tryptophan, glycerophospholipid, glyoxylate and dicarboxylate, lysine degradation, biosynthesis of folate, zeatin and lipopolysaccharide, degradation of nitrotoluene, benzoate, chlorocyclohexane and chlorobenzene, drug metabolism, catabolism of aromatic compounds, etc. (**Supplementary Figures S3** and **S4**). Our analysis demonstrates the microbial diversity and distribution of various metabolic pathways over a previously unexplored range of bacterial phyla.

**FIGURE 6:**
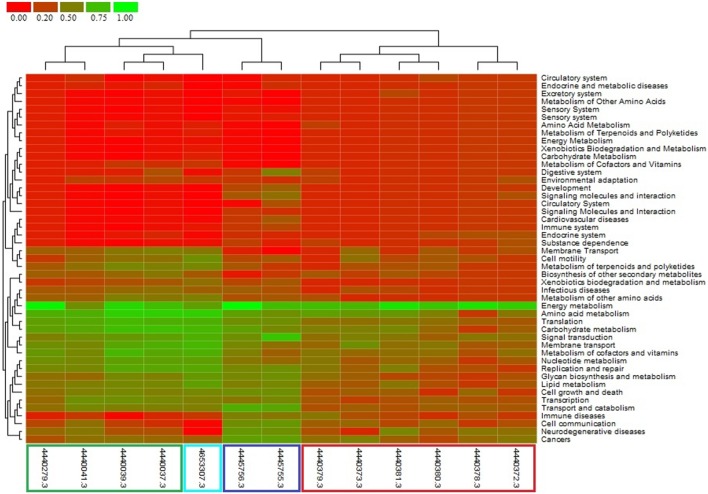
**Comparative functional profile of metagenomes generated using the KEGG (level 2) classification of reads on MG-RAST server.** Metagenome data source: this study (cyan color), Dinsdale et al., 2008 (green color), Vega Thurber et al., 2009 (red color), and Littman et al., 2011 (blue color).

**FIGURE 7:**
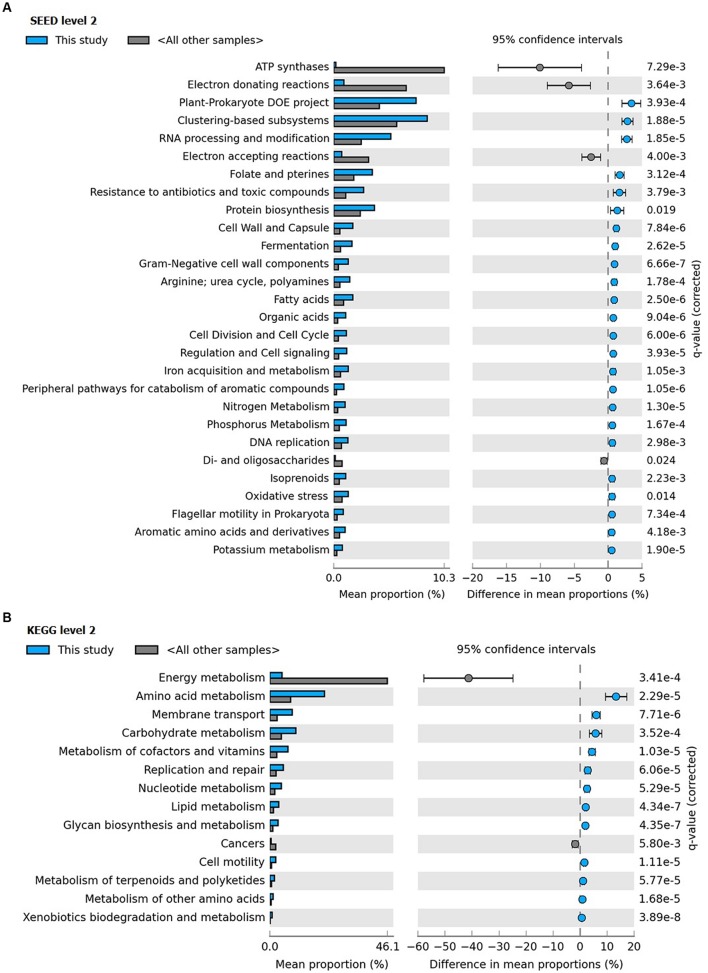
**Distribution of the (A) SEED (level 2) and (B) KEGG (level 2) categories with differences between the coral *F. echinata* metagenome (this study; cyan color) and other coral metagenomes (dark gray color)**.

Clustering of the metagenome of *F. echinata* based on relative abundance of functional genes with those of healthy corals from the Great Barrier Reef and the Northern Line Islands’ reefs (**Figure 6**, **Supplementary Figure S4**) suggested that the association between the coral host and bacterial community very likely mutualistic in nature. Further, statistical tests of the relative abundance of genes encoding functions related to various SEED (level 2) and KEGG (level 2) categories showed significantly (*p*-value <0.05) higher proportions of genes encoding functions affiliated to resistance to antibiotics and toxic compounds, iron acquisition and metabolism, potassium, phosphorous and nitrogen metabolism, RNA processing and modification, oxidative stress, DNA replication and repair, signal transduction, membrane transport, cell motility, cofactors and vitamins metabolism, carbohydrates, lipids and amino acids metabolism, glycan biosynthesis and metabolism. In contrast, the genes related to the functions of energy metabolism and respiration, such as ATP synthases, electron donating and accepting reactions, and metabolism of di- and oligosaccharides were detected at significantly (*p*-value <0.05) lower proportions compared to the other coral metagenomes (**Figure 7**).

These findings indicated that specific environmental stressors and niches promote enrichment of specific metabolic pathways and functions which provides adaptive advantages to the bacterial community associated with corals in oligotrophic seawater. The metabolic processes related to signal transduction, membrane transport, and cell motility play an important role in structuring of the microbial communities. The high proportion of genes associated with these functions in the *F. echinata* metagenome as compared to the other coral metagenomes indicated that the mucus-associated bacteria have the metabolic flexibility to adapt in the changing nutrient concentrations and chemical gradients in the coral micro-niche.

## Conclusions

The present study provides a metagenomic snapshot of the microbial community composition and functions in the mucus layer of the coral *F. echinata* from Andaman Sea. Our results showed an exclusive dominance of *Bacteria* affiliated to the class *Gammaproteobacteria* (specially, genus *Psychrobacter*, *Thalassolituus*, and *Cobetia*), while the virus-like sequences affiliated to the order *Caudovirales* constituted only a small fraction. Overall, the coral mucus-associated bacterial community was heterotrophic and carried genes required for the metabolism of complex organic compounds, such as proteins, lipids, and polysaccharides found in the coral mucus. Comparison with other coral metagenomes (both healthy and diseased corals) identified metabolic functions that are either required or advantageous for the coral colonization, such as resistance to antibiotics and toxic compounds, signal transduction, cell motility and membrane transport. Finally, mucus-associated microbiome of *F. echinata* is directly linked with local chemical gradients in the environment and different from those reported in other coral species from different geographic regions.

## Author Contributions

SD conceived the idea of the work. JB designed the experiments and performed the experiments. JB, TG, and SD analyzed the data and wrote the manuscript.

## Conflict of Interest Statement

The authors declare that the research was conducted in the absence of any commercial or financial relationships that could be construed as a potential conflict of interest.

## Footnotes

1 http://weizhong-lab.ucsd.edu/metagenomic-analysis/server/

2 https://metagenomics.anl.gov/
