# Supplementary material for: Composition and Functional Characterization of Microbiome Associated with Mucus of the Coral Fungia echinata Collected from Andaman Sea
Source: Front Microbiol. 2016 Jun 16;7:936. doi: 10.3389/fmicb.2016.00936 (PMC4909750; doi:10.3389/fmicb.2016.00936)
Supplement: Supplementary file 1 [file Data_Sheet_1.PDF]

Supplementary materials

Composition and functional characterization of microbiome associated with mucus of the coral *Fungia echinata* collected from Andaman Sea

Authors: Jhasketan Badhai[1], Tarini Shankar Ghosh[2] and Subrata K Das[1*]

Addresses: [1]Department of Biotechnology, Institute of Life Sciences, Nalco Square, Bhubaneswar, India, [2]Computational and Systems Biology group, Genome Institute of Singapore, 60 Biopolis St, Singapore.

Key words: Coral, Andaman Sea, metagenome, microbial composition, functional analysis.

[*]Corresponding author. Mailing address:
Institute of Life Sciences
Department of Biotechnology
Nalco Square, Bhubaneswar 751023 India
E-mail: subratkdas@hotmail.com / subrata@ils.res.in
Phone: (+91) 674 230 3342
Fax: (+91) 674 230 0728

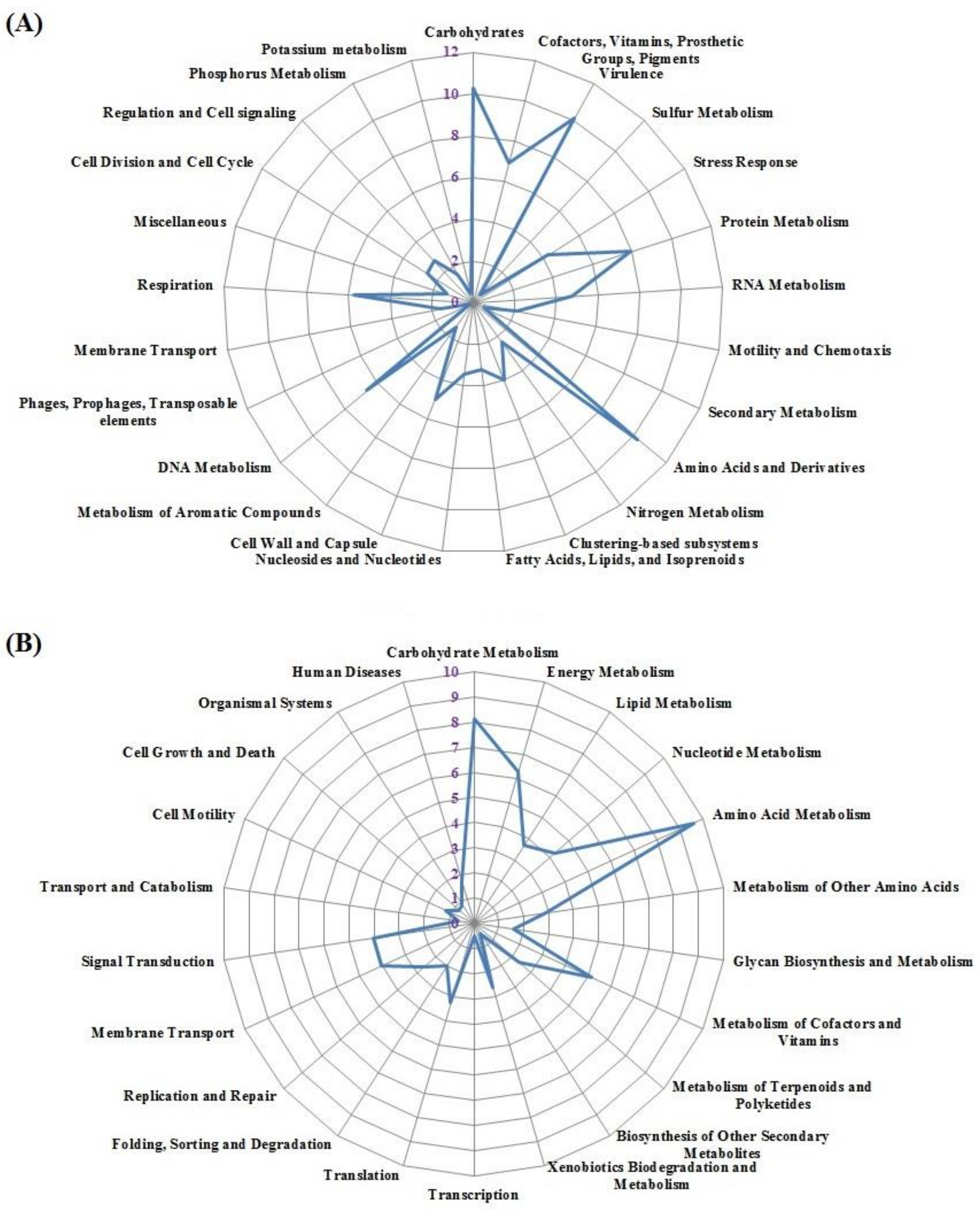

Supplementary Figure 1: Frequency distribution of genes related to different functional categories present in the metagenome based on (A), SEED subsystems and (B), KEGG category annotation. Number on each ring represents the percentage of genes in each functional category.

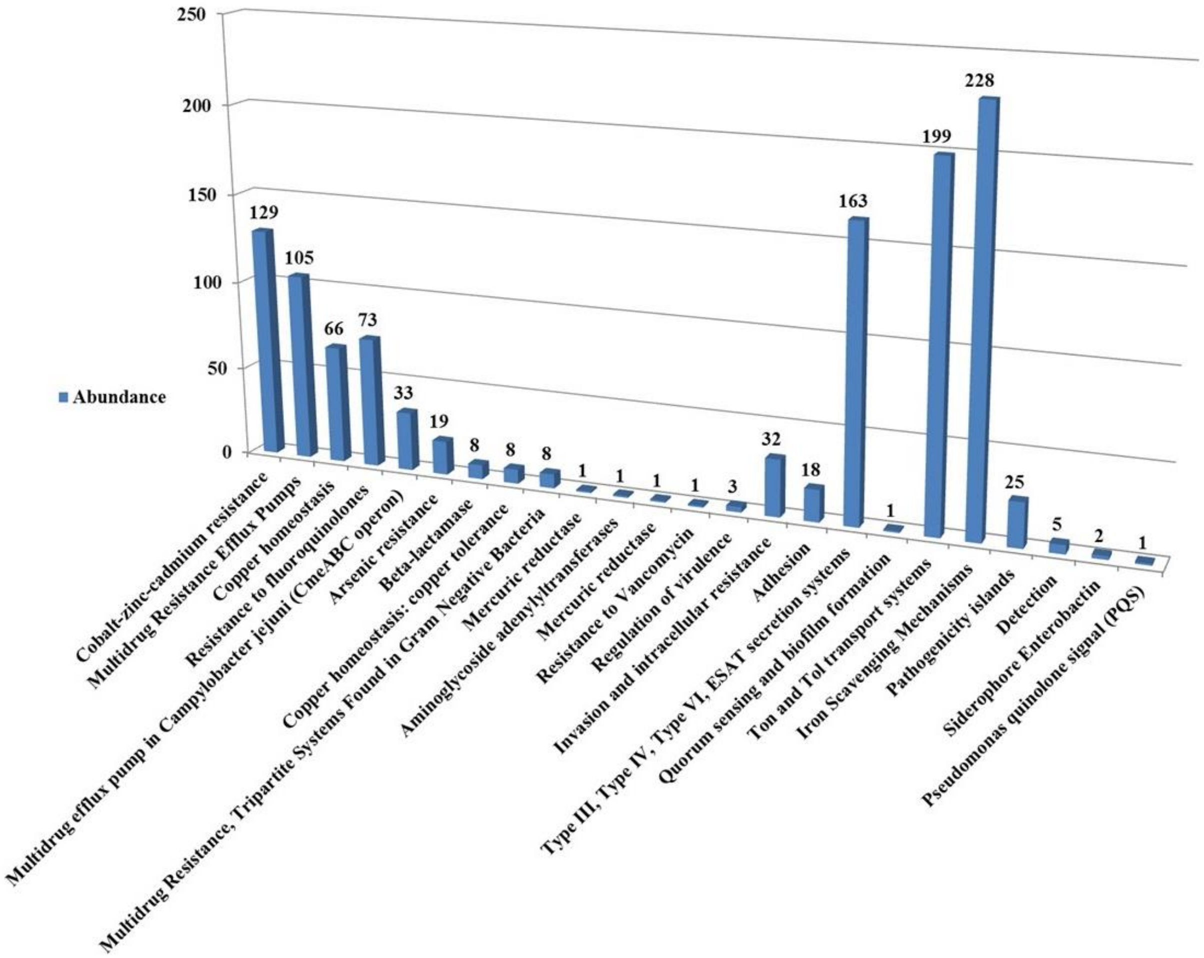

Supplementary Figure 2: Distribution of genes assigned to the SEED subsystem of virulence functions present in the metagenome. Numbers on top of the columns represent the gene count.

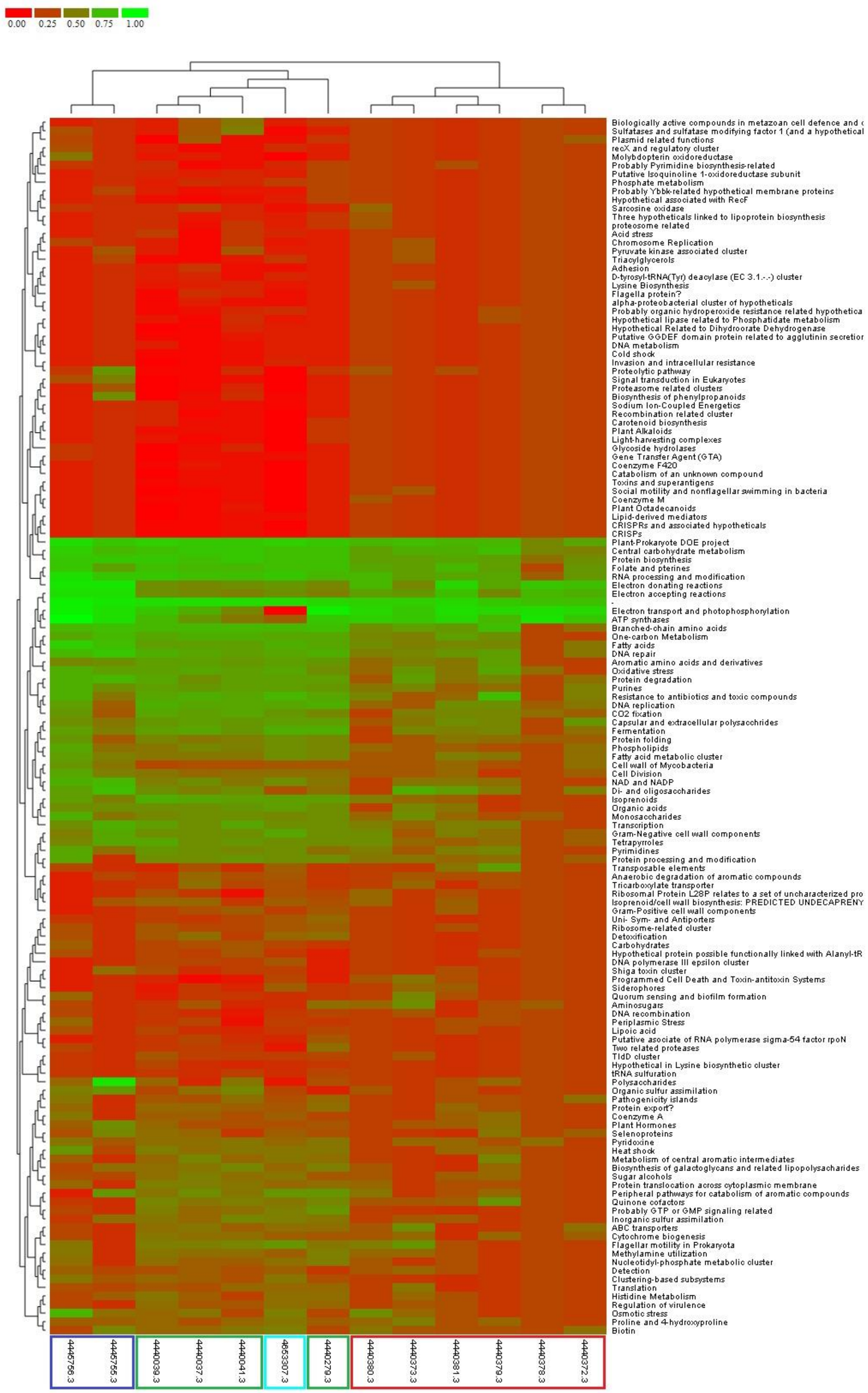
0.00
0.25
0.50
0.75
1.00
Biologically active compounds in metazoan cell defence and c
Sulfatases and sulfatase modifying factor 1 (and a hypothetical
Plasmid related functions
recX and regulatory cluster
Molybdopterin oxidoreductase
Probably Pyrimidine biosynthesis-related
Putative Isoquinoline 1-oxidoreductase subunit
Phosphate metabolism
Probably Ybbk-related hypothetical membrane proteins
Hypothetical associated with RecF
Sarcosine oxidase
Three hypotheticals linked to lipoprotein biosynthesis
proteosome related
Acid stress
Chromosome Replication
Pyruvate kinase associated cluster
Triacylglycerols
Adhesion
D-tyrosyl-tRNA(Tyr) deacylase (EC 3.1.-.-) cluster
Lysine Biosynthesis
Flagella protein?
alpha-proteobacterial cluster of hypotheticals
Probably organic hydroperoxide resistance related hypothetica
Hypothetical lipase related to Phosphatidate metabolism
Hypothetical Related to Dihydroorate Dehydrogenase
Putative GGDEF domain protein related to agglutinin secretior
DNA metabolism
Cold shock
Invasion and intracellular resistance
Proteolytic pathway
Signal transduction in Eukaryotes
Proteasome related clusters
Biosynthesis of phenylpropanoids
Sodium Ion-Coupled Energetics
Recombination related cluster
Carotenoid biosynthesis
Plant Alkaloids
Light-harvesting complexes
Glycoside hydrolases
Gene Transfer Agent (GTA)
Coenzyme F420
Catabolism of an unknown compound
Toxins and superantigens
Social motility and nonflagellar swimming in bacteria
Coenzyme M
Plant Octadecanoids
Lipid-derived mediators
CRISPRs and associated hypotheticals
CRISPs
Plant-Prokaryote DOE project
Central carbohydrate metabolism
Protein biosynthesis
Folate and pterines
RNA processing and modification
Electron donating reactions
Electron accepting reactions
-
Electron transport and photophosphorylation
ATP synthases
Branched-chain amino acids
One-carbon Metabolism
Fatty acids
DNA repair
Aromatic amino acids and derivatives
Oxidative stress
Protein degradation
Purines
Resistance to antibiotics and toxic compounds
DNA replication
CO2 fixation
Capsular and extracellular polysacchrides
Fermentation
Protein folding
Phospholipids
Fatty acid metabolic cluster
Cell wall of Mycobacteria
Cell Division
NAD and NADP
Di- and oligosaccharides
Isoprenoids
Organic acids
Monosaccharides
Transcription
Gram-Negative cell wall components
Tetrapyrroles
Pyrimidines
Protein processing and modification
Transposable elements
Anaerobic degradation of aromatic compounds
Tricarboxylate transporter
Ribosomal Protein L28P relates to a set of uncharacterized pro
Isoprenoid/cell wall biosynthesis: PREDICTED UNDECAPRENY
Gram-Positive cell wall components
Uni- Sym- and Antiporters
Ribosome-related cluster
Detoxification
Carbohydrates
Hypothetical protein possible functionally linked with Alanyl-tR
DNA polymerase III epsilon cluster
Shiga toxin cluster
Programmed Cell Death and Toxin-antitoxin Systems
Siderophores
Quorum sensing and biofilm formation
Aminosugars
DNA recombination
Periplasmic Stress
Lipoic acid
Putative asociate of RNA polymerase sigma-54 factor rpoN
Two related proteases
TldD cluster
Hypothetical in Lysine biosynthetic cluster
tRNA sulfuration
Polysaccharides
Organic sulfur assimilation
Pathogenicity islands
Protein export?
Coenzyme A
Plant Hormones
Selenoproteins
Pyridoxine
Heat shock
Metabolism of central aromatic intermediates
Biosynthesis of galactoglycans and related lipopolysacharides
Sugar alcohols
Protein translocation across cytoplasmic membrane
Peripheral pathways for catabolism of aromatic compounds
Quinone cofactors
Probably GTP or GMP signaling related
Inorganic sulfur assimilation
ABC transporters
Cytochrome biogenesis
Flagellar motility in Prokaryota
Methylamine utilization
Nucleotidyl-phosphate metabolic cluster
Detection
Clustering-based subsystems
Translation
Histidine Metabolism
Regulation of virulence
Osmotic stress
Proline and 4-hydroxyproline
Biotin
4445756.3
4445755.3
4440039.3
4440037.3
4440041.3
4653307.3
4440279.3
4440380.3
4440373.3
4440381.3
4440379.3
4440378.3
4440372.3

56

Supplementary Figure 3: Comparative functional profile of metagenomes generated using the SEED (level 2) classification of reads on MG-RAST server. Metagenome data source: this study (cyan colour), Dinsdale et al., 2008 (green colour), Vega Thurber et al., 2009 (red colour), and Littman et al., 2011 (blue colour).

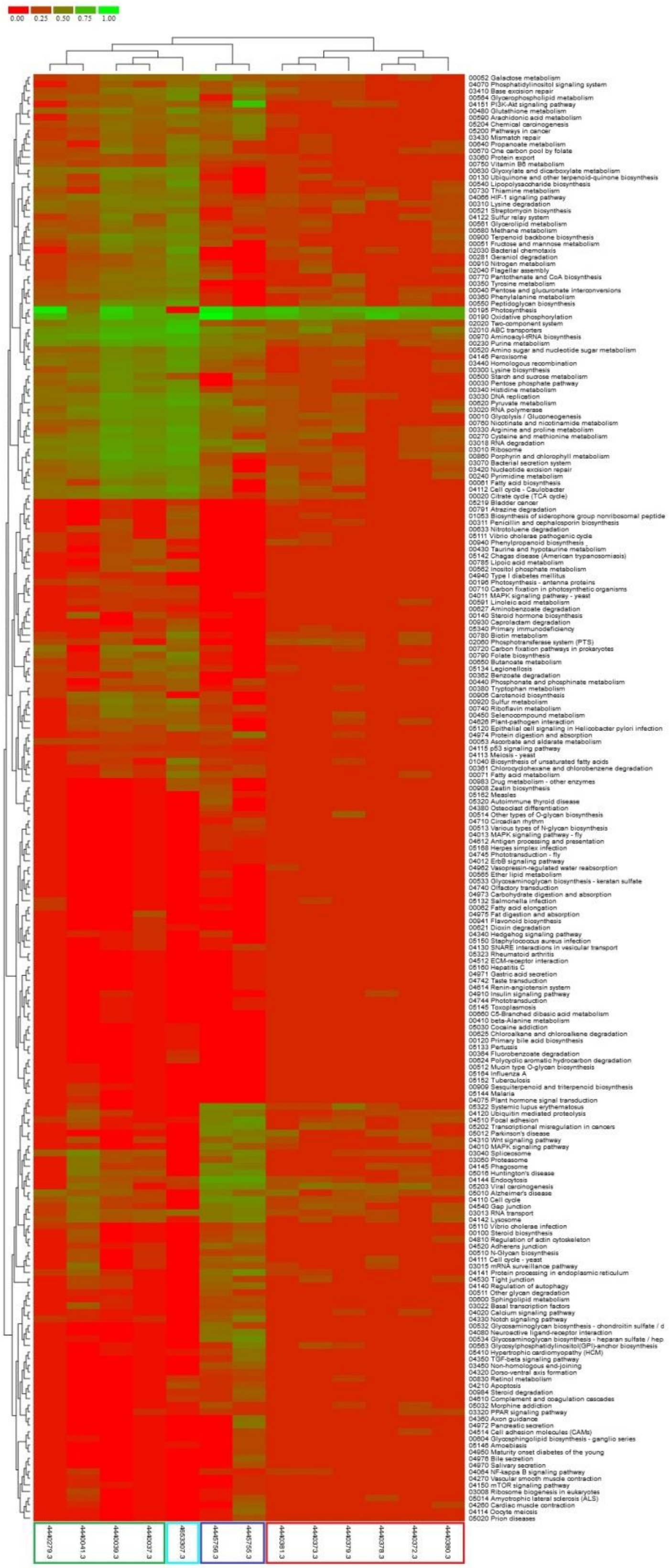

Supplementary Figure 4: Comparative functional profile of metagenomes generated using the KEGG (level 3) classification of reads on MG-RAST server. Metagenome data source: this study (cyan colour), Dinsdale et al., 2008 (green colour), Vega Thurber et al., 2009 (red colour), and Littman et al., 2011 (blue colour).
